# Attenuation of Eph Receptor Kinase Activation in Cancer Cells by Coexpressed Ephrin Ligands

**DOI:** 10.1371/journal.pone.0081445

**Published:** 2013-11-29

**Authors:** Giulia Falivelli, Erika Mathes Lisabeth, Elena Rubio de la Torre, Gizeh Perez-Tenorio, Giovanna Tosato, Ombretta Salvucci, Elena B. Pasquale

**Affiliations:** 1 Sanford-Burnham Medical Research Institute, San Diego, California, United States of America; 2 Laboratory of Cellular Oncology, Center for Cancer Research, National Cancer Institute, National Institutes of Health, Bethesda, Maryland, United States of America; 3 Department of Pathology, University of California San Diego, San Diego, California, United States of America; 4 Department Pharmacology, University of Bologna, Bologna, Italy; Rutgers University, United States of America

## Abstract

The Eph receptor tyrosine kinases mediate juxtacrine signals by interacting “in *trans*” with ligands anchored to the surface of neighboring cells via a GPI-anchor (ephrin-As) or a transmembrane segment (ephrin-Bs), which leads to receptor clustering and increased kinase activity. Additionally, soluble forms of the ephrin-A ligands released from the cell surface by matrix metalloproteases can also activate EphA receptor signaling. Besides these *trans* interactions, recent studies have revealed that Eph receptors and ephrins coexpressed in neurons can also engage in lateral “*cis*” associations that attenuate receptor activation by ephrins in *trans* with critical functional consequences. Despite the importance of the Eph/ephrin system in tumorigenesis, Eph receptor-ephrin *cis* interactions have not been previously investigated in cancer cells. Here we show that in cancer cells, coexpressed ephrin-A3 can inhibit the ability of EphA2 and EphA3 to bind ephrins in *trans* and become activated, while ephrin-B2 can inhibit not only EphB4 but also EphA3. The *cis* inhibition of EphA3 by ephrin-B2 implies that in some cases ephrins that cannot activate a particular Eph receptor in *trans* can nevertheless inhibit its signaling ability through *cis* association. We also found that an EphA3 mutation identified in lung cancer enhances *cis* interaction with ephrin-A3. These results suggest a novel mechanism that may contribute to cancer pathogenesis by attenuating the tumor suppressing effects of Eph receptor signaling pathways activated by ephrins in *trans*.

## Introduction

Members of the large Eph receptor tyrosine kinase family, and particularly EphA2 and EphB4, are overexpressed in a wide variety of tumor types [1,2]. Eph receptors signal by interacting “in *trans*” with ephrins expressed on neighboring cells, which promotes receptor clustering, autophosphorylation and kinase activity [3]. Soluble forms of the ephrin-A ligands released from the cell surface by matrix metalloproteases can also activate EphA receptors [4-7]. However, Eph receptors in cancer cells are often poorly tyrosine phosphorylated [3]. This suggests low activation by ephrin ligands and is consistent with the tumor suppressing effects reported for a number of Eph receptor downstream signaling pathways [1,8,9]. 

The lack of substantial Eph receptor activation is in some cases due to low expression of ephrin ligands in cancer cells with high receptor expression [1,10-13]. In addition, several other mechanisms can keep Eph receptor activation low in cancer cells that also express ephrin ligands. For example, cancer mutations have been shown to disrupt the ephrin binding ability or kinase activity of Eph receptors [14,15]. Furthermore, lack of E-cadherin-dependent cell-cell adhesion can impair EphA2 receptor activation in breast cancer cells, suggesting inefficient EphA2 *trans* interaction with ephrins [16]. Another potential mechanism to attenuate Eph receptor downstream signaling in cancer cells could involve inhibitory lateral *cis* interactions between Eph receptors and ephrins coexpressed in the same cells [2,17,18]. Inhibitory *cis* interactions with ephrins have been shown to play an important role in fine tuning Eph receptor activation in the nervous system to precisely control axon pathfinding and synaptic function [1,18-21]. However, *cis* interactions do not occur in all neurons coexpressing Eph receptors and ephrins because in some neurons receptors and ligands occupy distinct microdomains of the plasma membrane and thus cannot intermingle [20,22]. Whether *cis* interactions between Eph receptors and ephrins can also occur in cancer cells has not been previously investigated.

Biochemical and structural studies have shown that *cis* interaction involves an Eph receptor-ephrin binding interface distinct from that mediating the high affinity interaction in *trans* [18,23]. The extracellular region of both EphA and EphB receptor classes contains an N-terminal ligand-binding domain, a cysteine-rich region and two fibronectin type III domains [3]. The second fibronectin domain is followed by a transmembrane segment and a cytoplasmic region that includes the tyrosine kinase domain, a SAM domain and a PDZ-binding motif. The ephrins consist of an N-terminal Eph receptor-binding domain connected by a short linker region to a glycosylphosphatidylinositol (GPI) anchor for the ephrin-As and a transmembrane segment followed by a short cytoplasmic region for the ephrin-Bs. Eph receptor-ephrin binding in *trans* mainly involves the interaction between the G-H loop of the ephrin and a pocket within the ligand-binding domain of the Eph receptor [24]. These interfaces predominantly support the promiscuous interactions of Eph receptors with ephrins belonging to the same A or B class. On the other hand, *cis* interactions have been proposed to involve the fibronectin type III domains of the Eph receptor and a region of the receptor-binding domain of the ephrin that is distinct from the G-H loop [18,23]. 

Here we show that Eph receptors and ephrins coexpressed in cancer cells can engage in *cis* interactions that inhibit Eph receptor activation by ephrins in *trans*. Interestingly, we detected inhibition of EphA3 activation through *cis* interaction with not only ephrin-A3 but also ephrin-B2, which is not an activating ligand for EphA3 [25], suggesting that *cis* interactions do not exhibit the same receptor-ligand selectivity as *trans* interactions. We also found that a lung cancer mutation identified in the second fibronectin type III repeat of EphA3 enhances the *cis* association of the receptor with ephrin-A3. 

## Results

### Ephrin-A3 coexpression in cancer cells attenuates EphA receptor activation in trans by soluble ephrin-A3

**Figure 1:**
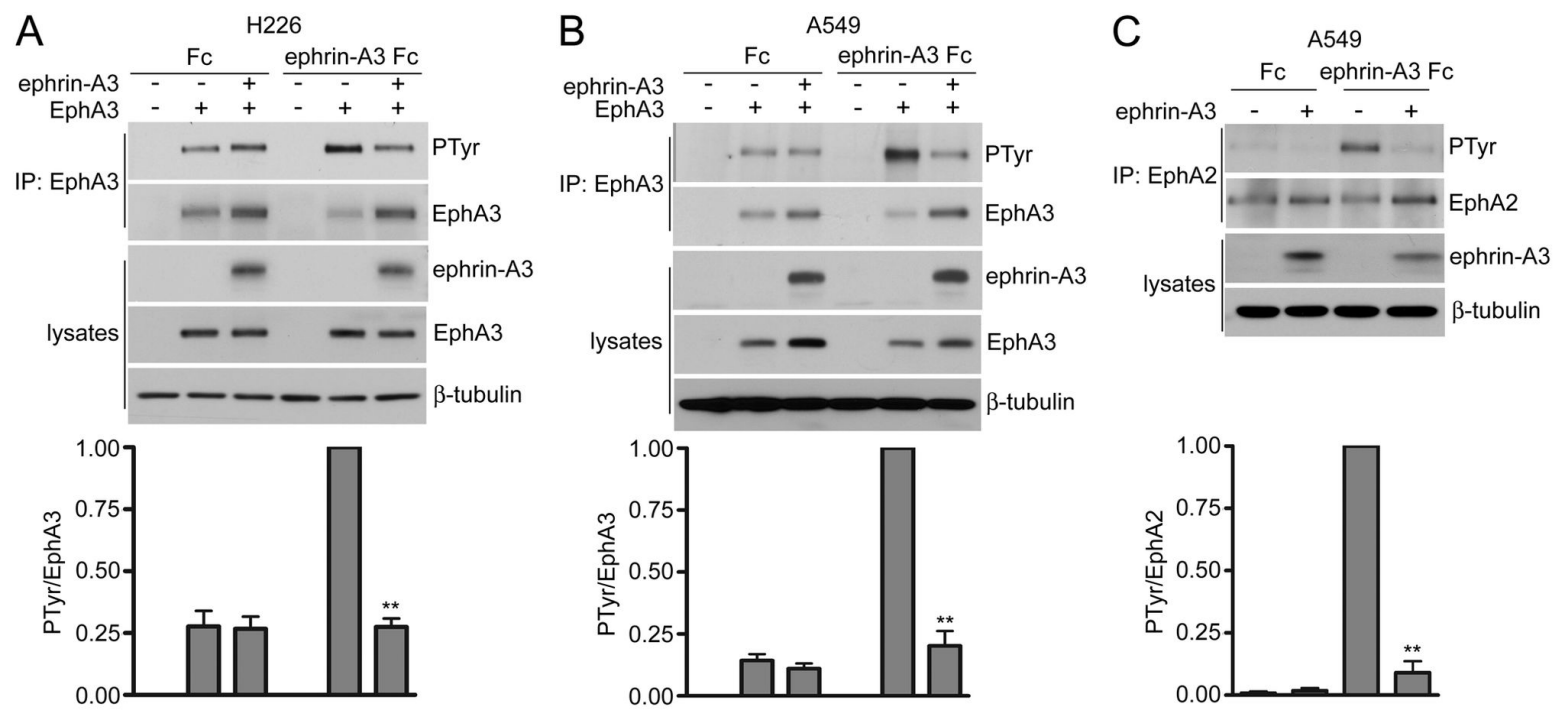
Coexpressed ephrin-A3 attenuates EphA receptor activation in cancer cells. (A,B) NCI-H226 and A549 lung cancer cells were infected with a lentivirus encoding EphA3 and ZsGreen alone or together with a lentivirus encoding mCherry-ephrin-A3; control cells were infected with lentiviruses encoding ZsGreen and mCherry. EphA3 immunoprecipitates were probed by immunoblotting for phosphotyrosine (PTyr) and reprobed for EphA3. Lysates were probed for mCherry-ephrin-A3 with an anti-dsRed antibody that also recognizes mCherry, for EphA3, and for β-tubulin as loading control. The histograms show normalized means ± SE quantified from 3 immunoblots in both A and B. In one of the A549 experiments used for quantification, the cells were stimulated with ephrin-A5 Fc. **p<0.01 by one sample t test for the comparison of ephrin-A3 Fc-treated cells expressing both EphA3 and ephrin-A3 with ephrin-A3 Fc-treated cells expressing only EphA3. Of note, EphA3 levels were higher in A549 cells co-expressing ephrin-A3/ephrin-B2 (see also Figs. 2A, 3B, 4A and 5C,D), suggesting that this receptor may be stabilized by the coexpressed ephrins. (C) A549 cells were infected with a lentivirus encoding mCherry-ephrin-A3 or mCherry as a control. Immunoprecipitated endogenous EphA2 was probed by immunoblotting for phosphotyrosine (PTyr) and reprobed for EphA2. Lysates were probed with an anti-dsRed antibody and β-tubulin as loading control. The histogram shows normalized means ± SE quantified from 3 immunoblots. **p<0.01 by one sample t test for the comparison of ephrin-A3 Fc-treated cells expressing or not expressing ephrin-A3.

To investigate the effect of ephrin coexpression on Eph receptor signaling in cancer cells, we examined EphA3 (an Eph receptor for which inhibitory *cis* interactions with ephrin-As have been extensively studied in neurons [17,18,20]) and EphA2 (the EphA receptor most widely expressed in cancer cells [1,26-28] but for which the effects of *cis* interactions were not previously investigated). We infected the NCI-H226 and A549 lung cancer cell lines with lentiviruses encoding EphA3 and ZsGreen from a bicistronic transcript or only ZsGreen as a control. After selection by FACS sorting, we further infected the cells with lentiviruses encoding ephrin-A3 tagged with mCherry or only mCherry as a control, followed by selection. The two lentivirally infected cancer cell lines, which do not express detectable endogenous EphA3 or ephrin-A3 (Figure 1), were then treated with ephrin-A3 Fc (a soluble form of the ephrin-A3 ligand fused to the Fc portion of human IgG_1_) to activate EphA3 through ephrin binding in *trans*. Ephrin-A3 Fc increased receptor tyrosine phosphorylation in the cells coexpressing EphA3 with control mCherry, as expected, but not in the cells coexpressing EphA3 with mCherry-ephrin-A3 (Figure 1A,B). Ephrin-A3 coexpression also attenuated ephrin-A3 Fc-induced activation of endogenous EphA2 in A549 cells (Figure 1C). Thus, in lung cancer cells, coexpressed ephrin-A3 can inhibit EphA2 and EphA3 activation by ephrin ligands.

### Coexpression with ephrin-A3 in cancer cells impairs the ability of EphA3 to bind ephrin-As in trans

**Figure 2:**
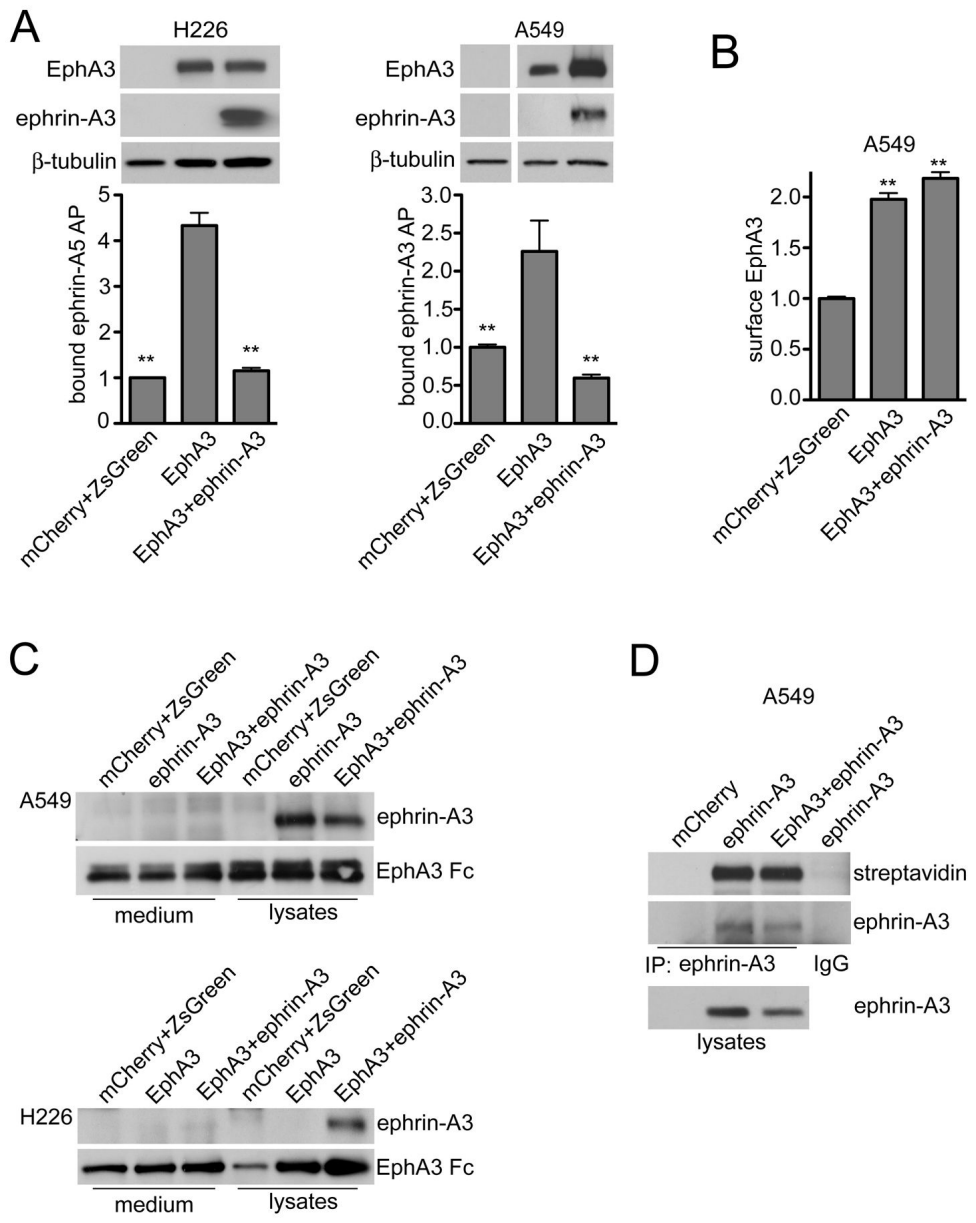
Coexpressed cell surface-associated ephrin-A3 inhibits the binding in *trans* of soluble ephrins to EphA3 in lung cancer cells. (A) NCI-H226 and A549 lung cancer cells were infected with a lentivirus encoding EphA3 and ZsGreen alone or together with a lentivirus encoding mCherry-ephrin-A3; control cells were infected with lentiviruses encoding ZsGreen and mCherry. The histograms show the binding of ephrin-A5 AP to NCI-H226 cells and ephrin-A3 AP to A549 cells, revealing that ephrin-A3 coexpression prevents the binding of ephrin AP proteins to EphA3. Normalized means from 2 experiments (each with triplicate samples) ± SE are shown. **p<0.01 by one-way ANOVA and Dunnett’s post-hoc test for the comparison with cells expressing only EphA3. The immunoblots show expression of EphA3, ephrin-A3, and β-tubulin as loading control in cell lysates, verifying that ephrin-A3 coexpression did not reduce EphA3 levels. In fact, EphA3 levels appeared higher in A549 cells co-expressing ephrin-A3. The white space indicates removal of an irrelevant lane. (B) Cell surface biotinylation followed by an ELISA where EphA3 was captured with an immobilized antibody and its biotinylation detected with streptavidin-HRP reveals a similar fraction of EphA3 on the surface of cells expressing EphA3 alone or together with ephrin-A3. The histogram shows means from 2 experiments (each with triplicate samples) ± SE. Incubation with twice as much lysates yielded similar results, indicating that maximal EphA3 binding to the antibody immobilized in the wells was achieved. **p<0.01 by one-way ANOVA and Tukey’s post-hoc test for the comparison with cells expressing mCherry and ZsGreen; p>0.05 for the comparison of cells expressing EphA3 with and without ephrin-A3. (C) EphA3 Fc was used for pull-downs from conditioned medium and lysates of A549 or H226 cells infected with the indicated lentiviruses. By immunoblotting with an anti-dsRed antibody, ephrin-A3 was detected only in the lysates. The pull-downs were also probed for Fc to verify the levels of EphA3 Fc. (D) Surface proteins were biotinylated in cells infected with lentiviruses encoding mCherry, mCherry-ephrin-A3, or mCherry-ephrin-A3 together with EphA3 and ZsGreen. mCherry-ephrin-A3 immunoprecipitates (with anti-dsRed antibody) were probed with streptavidin-HRP, demonstrating similar cell surface levels of ephrin-A3 expressed alone or together with EphA3. IgG, control immunoprecipitate with non-immune IgGs. Lysates were probed for mCherry-ephrin-A3 with anti-dsRed antibody.

To examine whether in cancer cells ephrin-A3 coexpression impairs the ability of EphA3 to bind ephrin-A ligands in *trans*, we measured the binding of soluble forms of ephrin-A5 or ephrin-A3 fused to alkaline phosphatase (AP) to NCI-H226 and A549 cells expressing EphA3 alone or together with mCherry-ephrin-A3. We detected ephrin-A AP binding to cells only expressing EphA3 but not to cells coexpressing ephrin-A3 with EphA3 (Figure 2A). Immunoblotting verified that ephrin-A3 coexpression does not decrease overall EphA3 levels (Figure 2A). Biotinylation of cell surface proteins followed by an ELISA in which EphA3 was captured with an antibody and its level of biotinylation was detected with streptavidin conjugated to horseradish peroxidase (HRP) showed that ephrin-A3 coexpression does not affect the fraction of EphA3 present on the cell surface (Figure 2B). Thus, coexpressed ephrin-A3 in lung cancer cells inhibits ephrin binding to EphA3 in *trans* without reducing EphA3 expression or surface localization.

 A possible explanation for these results could be that soluble ephrin-A3 released in the culture medium by matrix metalloproteases [4-6] would compete with ephrin-A3 AP for binding to the EphA3 ligand-binding domain. To address this possibility, we used the extracellular domain of EphA3 fused with Fc to pull-down ephrin-A3 from the culture medium or the cells lysed in a volume equivalent to that of the culture medium. Ephrin-A3 could be detected by immunoblotting in the pull-downs from cell lysates but not from the culture medium (Figure 2C), indicating that the great majority of the ephrin-A3 remained associated with the cells during the 24-48 hour time period of our experiments. In addition, a single mCherry-ephrin-A3 band was observed in the immunoblots, making it unlikely that a substantial portion of the ephrin was cleaved to generate a smaller form remaining associated with the cells by binding to an EphA receptor. Biotinylation of cell surface proteins followed by detection of the immunoprecipitated biotinylated ephrin-A3 with streptavidin-HRP confirmed that ephrin-A3 is similarly localized on the A549 cell surface when expressed alone or together with EphA3 (Figure 2D). 

### EphA3-ephrin-A3 cis interaction does not require the receptor ligand-binding domain

**Figure 3:**
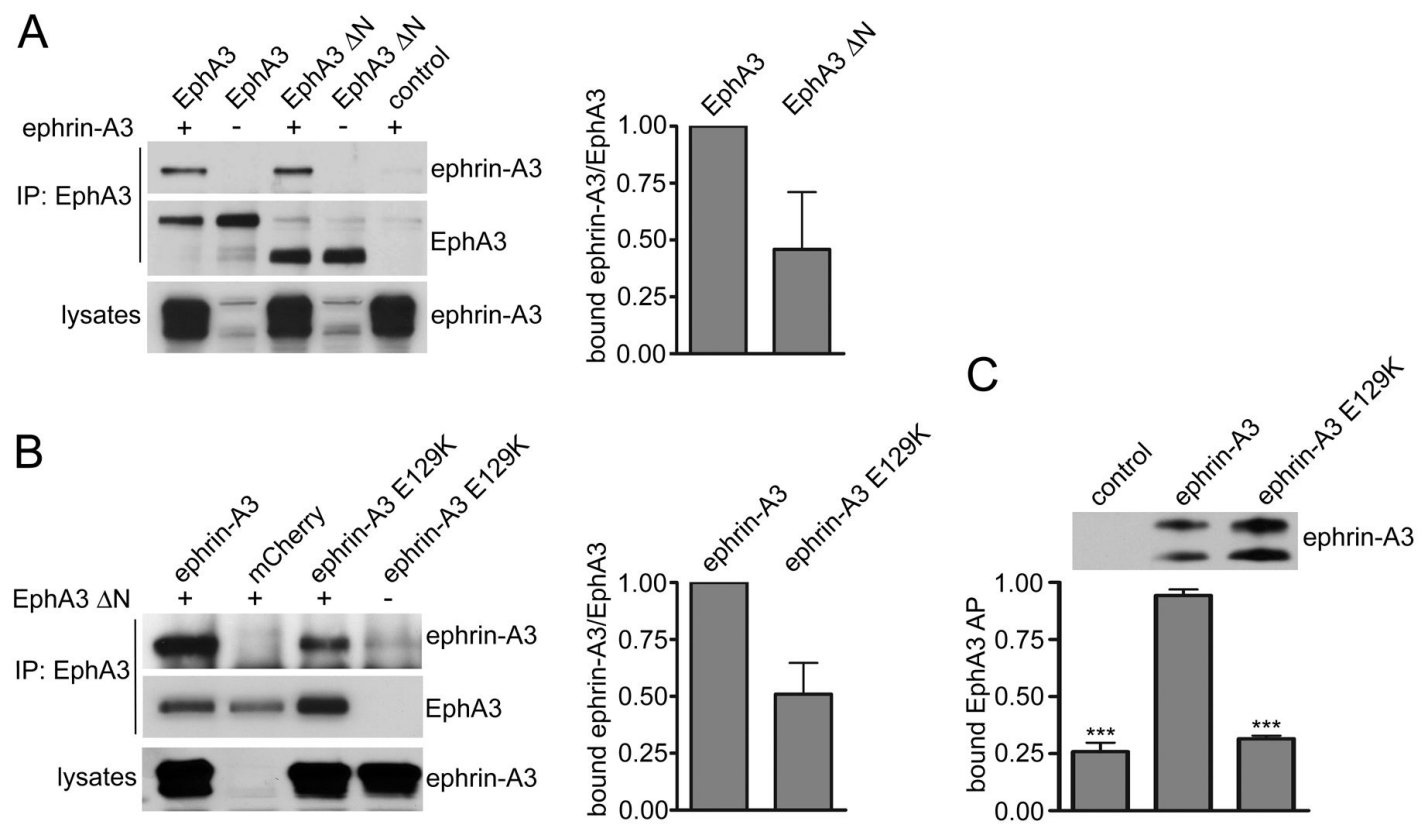
*Cis* interaction between coexpressed EphA3 and ephrin-A3 does not require the regions involved in trans interaction. (A) HEK AD-293 cells were infected with a lentivirus encoding mCherry-ephrin-A3 or mCherry as a control. Subsequently, the cells were transfected with plasmids encoding full-length EphA3 or a truncated form lacking the ligand-binding domain and cysteine-rich region (EphA3 ΔN). EphA3 immunoprecipitates were probed with anti-ephrin-A3 antiserum and reprobed for EphA3, revealing that ephrin-A3 association with EphA3 does not require the EphA3 ligand-binding domain. The histogram shows normalized means ± SE quantified from the immunoblots from 2 experiments. p>0.05 by one sample t test for the comparison of ephrin-A3 bound to EphA3 ΔN or full-length EphA3. (B) HEK AD-293 cells infected with a lentivirus encoding mCherry-ephrin-A3, the mCherry-ephrin-A3 E129K mutant, or mCherry as a control, were transfected with a plasmid encoding EphA3 ΔN. EphA3 immunoprecipitates were probed for ephrin-A3 and reprobed for EphA3, revealing that the E129K mutation does not abolish the *cis* interaction with EphA3. The histogram shows normalized means ± SE quantified from 3 immunoblots. p>0.05 by one sample t test for the comparison of ephrin-A3 E129K versus ephrin-A3 wild-type bound to EphA3 ΔN. (C) HEK AD-293 cells were transfected with control pcDNA3, pcDNA3-ephrin-A3, or pcDNA3-ephrin-A3 E129K. The histogram shows means from two experiments for the binding of EphA3 AP to ephrin-A3, confirming that ephrin-A3 E129K mutant does not bind EphA3 in *trans*. ***p<0.001 by one-way ANOVA and Dunnett’s post-hoc test for the comparison with cells expressing wild-type ephrin-A3. The immunoblot shows the expression of ephrin-A3 and ephrinA3 E129K in lanes loaded with equal amounts of total lysates. It should be noted that ephrin-A3 overexpressed in HEK cells yields two bands, with the upper band corresponding to the size of the mature full-length protein.

Previous studies have shown that cis interactions require membrane localization of the Eph receptor and the ephrin [18]. Therefore, to examine whether the EphA3 ligand-binding domain is necessary for *cis* interaction with ephrin-A3 or whether the fibronectin type III repeats are sufficient to mediate *cis* binding [18,23], we transiently transfected HEK293 cells stably expressing mCherry-ephrin-A3 with plasmids encoding EphA3 ΔN (a truncated form of EphA3 that lacks the N terminal ligand-binding domain and cysteine-rich region) or full-length EphA3. In coimmunoprecipitation experiments with an anti-EphA3 antibody that recognizes the C-terminal region of the receptor, we detected association of ephrin-A3 with both full-length and truncated EphA3 (Figure 3A). This confirms that the EphA3 ligand-binding domain, which mediates high affinity binding in *trans*, is not necessary for EphA3-ephrin-A3 *cis* interaction. 

To investigate the effect of mutating the ephrin G-H loop, we examined the E129K mutation in ephrin-A3. This mutation did not prevent the *cis* association of ephrin-A3 with EphA3 ΔN (Figure 3B), even though it abolished the *trans* interaction with EphA3 AP (Figure 3C). These results are consistent with those obtained with the corresponding ephrin-A5 E129K mutant, which can also still attenuate through *cis* interaction EphA3 phosphorylation as well as EphA-mediated growth cone collapse and axon guidance triggered by ephrin-A ligands in *trans* [18,20]. Hence, EphA3 and ephrin-A3 can associate with each other even when lacking the regions that mediate high affinity binding in *trans*, supporting the general involvement in *cis* interactions of the Eph fibronectin type III domains and an ephrin region distinct from the G-H loop. 

### The EphA3 G518L lung cancer mutation enhances cis interaction with coexpressed ephrin-A3

**Figure 4:**
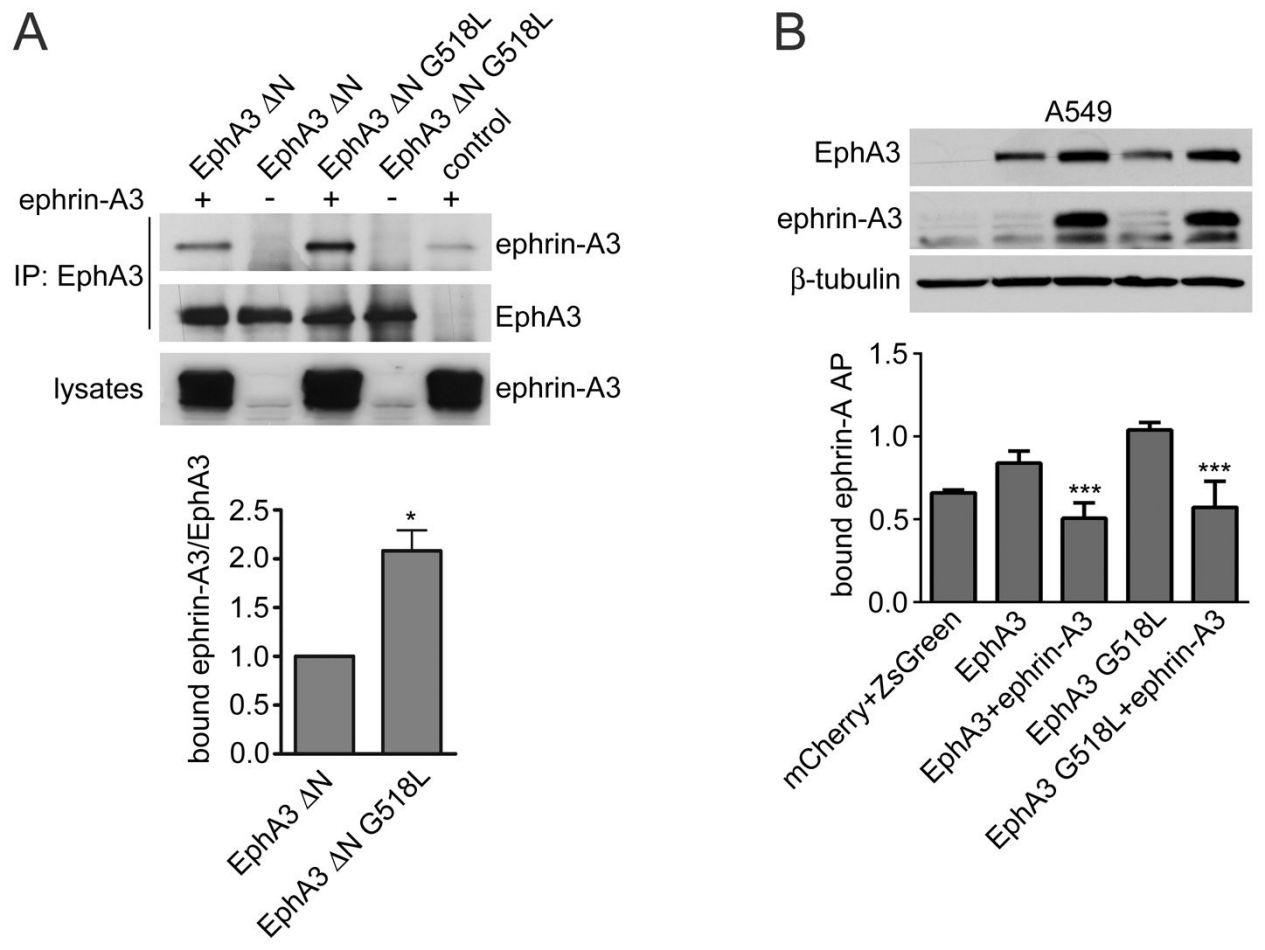
The EphA3 G518L lung cancer mutation enhances *cis* interaction with ephrin-A3. (A) HEK AD-293 cells were infected with a lentivirus encoding mCherry-ephrin-A3 or mCherry as a control. The cells were then transfected with EphA3 ΔN or the EphA3 ΔN G518L mutant. EphA3 immunoprecipitates were probed with an anti-ephrinA3 antiserum and reprobed for EphA3. The EphA3 G518L mutation found in lung cancer increases the affinity of the lateral interaction between EphA3 and ephrin-A3. The histogram shows normalized means ± SE quantified from the immunoblots from 3 experiments. *p<0.05 by one sample t test for the comparison of the EphA3 ΔN G518 mutant versus EphA3 ΔN. (B) A549 lung cancer cells were infected with a lentivirus encoding EphA3 wild-type or the G518L mutant and ZsGreen alone or together with a lentivirus encoding mCherry-ephrin-A3; control cells were infected with lentiviruses encoding ZsGreen and mCherry. The histogram shows cell binding of ephrin-A3 AP (one experiment) and ephrin-A5 AP (2 experiments), confirming that ephrin-A3 coexpression prevents the binding of ephrin AP proteins to the EphA3 G518L mutant. Normalized means from 3 experiments (each with duplicate samples) ± SE are shown. ***p<0.001 by one-way ANOVA and Tukey’s post-hoc test for the comparison of cells coexpressing EphA3 and ephrin-A3 with cells only expressing EphA3 and for the comparison of cells coexpressing EphA3 G518L and ephrin-A3 with cells only expressing EphA3 G518L. The immunoblot of the cell lysates shows expression of EphA3, ephrin-A3, and β-tubulin as loading control.

Recent sequencing studies have identified EphA3 mutations in lung cancer and other cancers, and functional characterization has revealed that many are loss-of-function mutations that inhibit ephrin binding, kinase activity and/or cell surface localization, suggesting a tumor suppressor role for wild-type EphA3 [14,15,29]. One of the few mutations that were not found to impair any of the EphA3 properties examined in a previous study, but rather slightly increased EphA3 cell surface localization, is the G518L mutation in the second fibronectin type III domain [14]. Since G518 in EphA3 corresponds to a conserved residue that in the EphA2-ephrin-A5 crystal structure participates in the *cis* interface, we examined whether the G518L mutation might affect the *cis* association of EphA3 with coexpressed ephrin-A3. To focus on the role of the cis interaction, we used EphA3 ΔN or the EphA3 ΔN G518L mutant. Coimmunoprecipitation experiments using HEK293 cells coexpressing mCherry-ephrin-A3 with EphA3 ΔN or the ΔN G518L mutant revealed more ephrin-A3 associated with the mutant (Figure 4A). Measurement of ephrin-A5 AP binding verified that ephrin-A3 co-expression with the full-length EphA3 G518 mutant inhibited its ability to bind ephrins in trans (Figure 4B). These results suggest that the G518L mutation enhances EphA3-ephrin binding in *cis* and supports the involvement in the *cis* interface of the conserved glycine in the second fibronectin type III domain [23].

### Ephrin-B2 coexpression in cancer cells attenuates not only EphB4 but also EphA3 activation and ligand-binding capacity in trans

**Figure 5:**
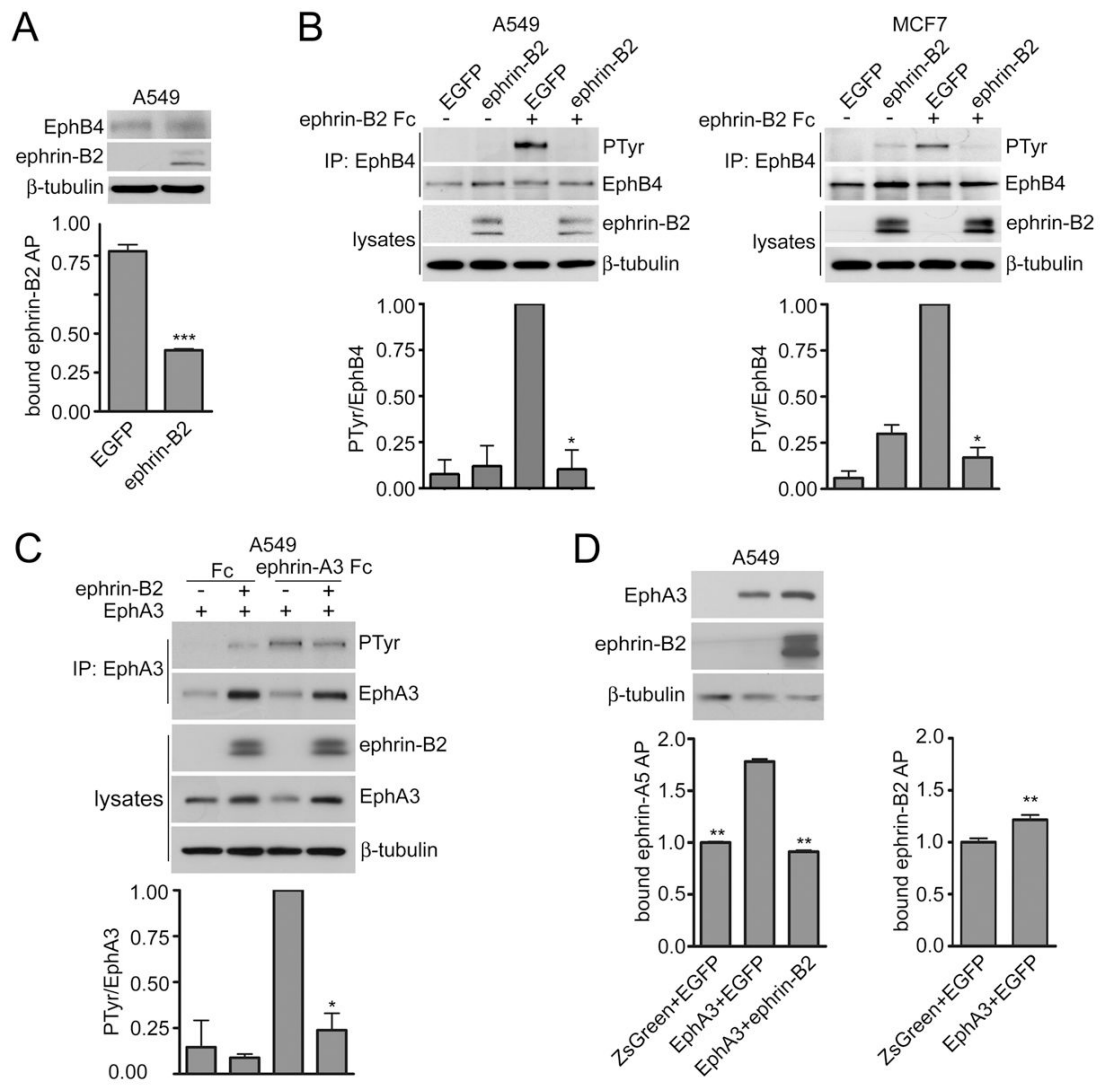
Coexpressed ephrin-B2 attenuates EphB4 as well as EphA3 activation in cancer cells. (A) The histogram shows the binding of ephrin-B2 AP to A549 cells infected with lentiviruses encoding EGFP-ephrin-B2 or EGFP, revealing that ephrin-B2 coexpression inhibits ephrin-B2 AP binding to EphB4. Normalized means from 3 experiments (each with triplicate samples) ± SE are shown. ***p<0.001 by unpaired t test for the comparison of cells expressing ephrin-B2 with cells not expressing ephrin-B2. The immunoblot of the cell lysates shows expression of EphB4, ephrin-B2 and β-tubulin as loading control. (B) A549 lung cancer cells and MCF7 breast cancer cells were infected with lentiviruses encoding EGFP-ephrin-B2 or EGFP. EphB4 immunoprecipitates were probed by immunoblotting for phosphotyrosine (PTyr) and reprobed for EphB4. Cell lysates were probed for ephrin-B2 with an anti-EGFP antibody and for β-tubulin as loading control. The histograms show normalized means ± SE quantified from 2 immunoblots for each cell line. *p<0.05 by one sample t test for the comparison of ephrin-B2 Fc-treated cells expressing ephrin-B2 with cells not expressing ephrin-B2. (C) A549 cells were infected with a lentivirus encoding EphA3 and ZsGreen together with a lentivirus encoding EGFP-ephrin-B2 or EGFP only. Control cells were infected with lentiviruses encoding ZsGreen and EGFP. EphA3 immunoprecipitates were probed by immunoblotting for phosphotyrosine (PTyr) and reprobed for EphA3. Lysates were probed for ephrin-B2 with an anti-EGFP antibody as well as for EphA3 and for β-tubulin as loading control. The histogram shows normalized means ± SE quantified from 2 immunoblots. *p<0.05 by one sample t test for the comparison of ephrin-A3 Fc-treated cells expressing ephrin-B2 with cells not expressing ephrin-B2. (D) Ephrin-A5 AP binding to cell surface EphA3 is inhibited by ephrin-B2 coexpression. The histogram shows means ± SE from 3 experiments (each with triplicate samples) for the binding of ephrin-A5 AP or ephrin-B2 AP to the A549 cells used for the experiment in C. For ephrin-A5 binding, **p<0.01 by one-way ANOVA and Dunnett’s post-hoc test for the comparison with cells expressing EphA3 and EGFP; for ephrin-B2 AP binding, **p<0.01 by unpaired t test for the comparison of cells expressing or not expressing EphA3. The immunoblot of the cell lysates shows expression of ephrin-B2, EphA3 and β-tubulin as loading control, verifying that ephrin-B2 coexpression did not reduce EphA3 levels. Of note, the doublet corresponding to overexpressed ephrin-B2 is not due to different degrees of N-linked glycosylation because removal of N-linked oligosaccharides with the PNGase-F endoglycosidase similarly increased the SDS-PAGE mobility of both bands (not shown). Whether the upper band may represent a form with O-linked oligosaccharides [51] or other posttranslational modification remains to be determined.

*Cis* interactions between the Eph fibronectin type III domains and ephrins could have distinctive selectivity compared to *trans* interactions involving the Eph ligand-binding domain and the ephrin G-H loop [23]. To investigate this, we used ephrin-B2, which does not bind with high affinity to the EphA3 ligand-binding domain [25]. We infected A549 lung cancer cells and MCF7 breast cancer cells with a lentivirus encoding ephrin-B2 fused to EGFP and first examined the effects on endogenous EphB4, which binds the ephrin-B2 ligand in *trans*. Like EphA2, EphB4 is widely expressed in cancer cells [1,30] and its ability to be regulated by ephrins in *cis* was not previously examined. We found that ephrin-B2 expression inhibits the binding of ephrin-B2 AP to the cell surface (Figure 5A) and EphB4 tyrosine phosphorylation induced in *trans* by ephrin-B2 Fc (Figure 5B). Thus, *cis* interaction with coexpressed ephrin-B2 inhibits EphB4 ligand binding in *trans* and activation in cancer cells. To examine whether EphA3 can also be regulated by *cis* interaction with ephrin-B2, we infected A549 lung cancer cells expressing EphA3 with lentiviruses encoding EGFP-ephrin-B2 or only EGFP as a control. Interestingly, ephrin-B2 coexpression attenuated EphA3 activation by ephrin-A3 Fc (Figure 5C) and inhibited the ability of EphA3 to bind ephrin-A5 AP without decreasing overall EphA3 levels (Figure 5D). EphA3 expression only slightly increased the binding of the extracellular domain of ephrin-B2 AP to the cells (Figure 5D), confirming that ephrin-B2 does not efficiently bind to EphA3 in *trans* [25]. These results suggest that although ephrin-B2 is not an activating ligand for EphA3, it can affect EphA3 function through *cis* interaction. This implies that the binding specificities that govern *cis* and *trans* Eph receptor-ephrin interactions are not the same. 

### Endogenous ephrin-As attenuate activation of coexpressed EphA2 in cancer cells

**Figure 6:**
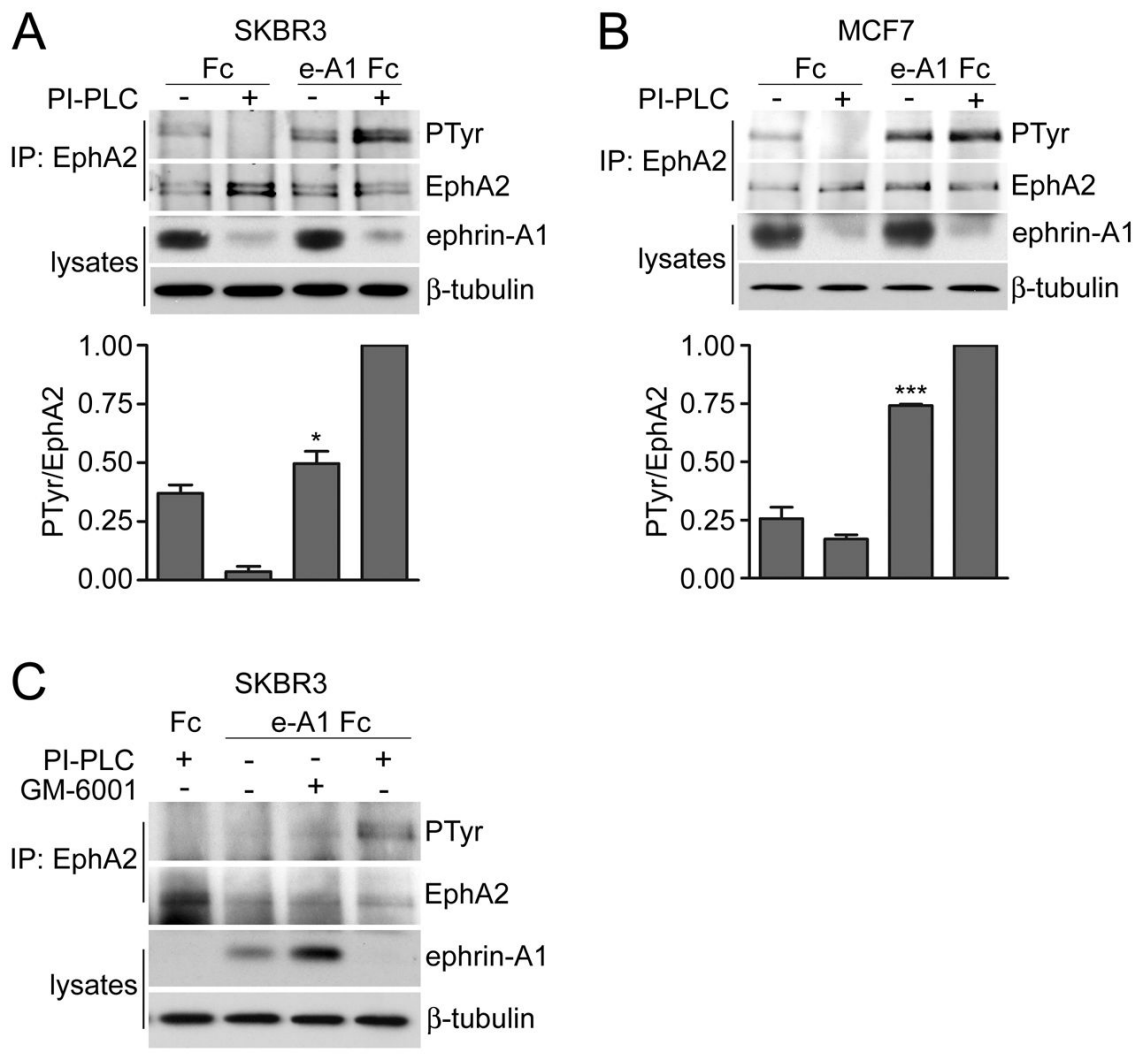
Removal of endogenous ephrin-As from the cell surface potentiates EphA2 activation by soluble ephrin-A1 in *trans*. (A) SKBR3 and (B) MCF7 breast cancer cells were treated with PI-PLC for 4 hours and then stimulated with ephrin-A1 Fc. EphA2 immunoprecipitates were probed by immunoblotting for phosphotyrosine (PTyr) and reprobed for EphA2. Lysates probed with anti-ephrin-A1 antibody verify removal of ephrin-As by PI-PLC; β-tubulin verifies equal loading of the lanes. The Odyssey LI-COR system was used for detection and the color images were converted to greyscale with Photoshop. The histograms show the normalized data from 3 different experiments *p<0.05 and ***p<0.001 by one sample t test for the comparison of ephrin-A1 Fc-stimulated cells treated or not with PI-PLC. (C) SKBR3 cells were treated with PI-PLC as in A or with the broad-spectrum matrix metalloprotease inhibitor GM-6001 for 24 hours. Immunoprecipitates and lysates were probed as indicated.

To investigate whether ephrins endogenously expressed in cancer cells can also engage in *cis* interactions that inhibit the activation of coexpressed endogenous Eph receptors, we chose the SKBR3 and MCF7 breast cancer cell lines. These lines express high levels of ephrin-A ligands together with EphA2 [11] (broadinstitute.org/ccle), although the receptor is expressed at relatively low levels, consistent with the complementary expression of Eph receptors and ephrins observed in many cancer cell lines [1]. Since both SKBR3 and MCF7 cells express multiple ephrin-A ligands, which are GPI-anchored, we used the enzyme phosphatidylinositol-specific phospholipase C (PI-PLC) to remove all ephrin-As from the cell surface. In both cell lines, removal of endogenous ephrin-As from the cell surface resulted in enhanced EphA2 activation by ephrin-A1 Fc in *trans* compared to untreated cells (Figure 6 A,B). In contrast, PI-PLC treatment of control Fc-treated cells decreased the low basal EphA2 activation, suggesting that endogenous ephrin-As can induce some EphA2 activation. Since ephrin-A1 has been reported to be cleaved from the surface of cancer cells by matrix metalloproteases, we also treated SKBR3 cells with the broad-spectrum matrix metalloprotease inhibitor GM-6001 [4,6,31]. Treatment with the inhibitor for 24 hours further increased cell surface associated ephrin-A1. However, it did not substantially affect EphA2 tyrosine phosphorylation induced by ephrin-A1 Fc binding in *trans*, possibly due to already high *cis* inhibition by the high levels of ephrin-A1 present even in the absence of GM-6001. Thus, in cancer cells *cis* interaction with endogenous ephrin-A ligands can attenuate EphA2 activation by ephrin-As presented in *trans*, supporting the significance of *cis* interactions in cancer pathogenesis.

## Discussion

Different families of receptors and cell surface-associated ligands that together mediate juxtacrine signals by interacting in *trans* across cell-cell junctions can also, when coexpressed on the same cell surface, interact laterally in *cis* [32]. These *cis* interactions, which have been mostly studied in the nervous system and the immune system, typically attenuate the signals triggered by the *trans* interactions through mechanisms that in many cases are not well understood [32-34]. Recent studies have uncovered key functional roles for inhibitory *cis* interactions between Eph receptors and ephrin ligands coexpressed in neurons [17-21]. However, despite the importance of the Eph/ephrin system in cancer pathogenesis, Eph receptor-ephrin *cis* interactions have not yet been investigated in cancer cells.

We have detected inhibitory *cis* interactions with ephrins in cancer cells not only for EphA3, which had been previously studied in neurons, but also for endogenous EphA2 and EphB4, for which the effects of *cis* interactions have not been previously investigated. Among the Eph receptors, EphA2 and EphB4 are the most widely expressed in epithelial and cancer cells, although most other Eph receptors including EphA3 are also aberrantly expressed in at least some cancers [1,35-39]. *Cis* interactions between coexpressed Eph receptors and ephrins may represent one of the strategies adopted by cancer cells to escape the tumor suppressing effects of Eph receptor signaling induced by ephrins binding in *trans*, including inhibition of cell growth and invasiveness [1,9,35,40-43].

We found that in cancer cells *cis* interactions can inhibit ephrin binding to Eph receptors in *trans*, consistent with previous studies in other systems [17,20]. This effect, which likely explains the observed inhibition of Eph receptor activation by ephrins *in trans*, could be due to different underlying mechanisms. We have shown that the levels of EphA3 on the cancer cell surface are not decreased by coexpression of ephrin-A3. We have also excluded occupancy of the EphA3 ligand-binding domain by ephrin-A3 that may be released into the medium by proteases [6]. Another possible mechanism by which *cis* interactions could lead to inhibition of the binding of soluble ephrins in *trans* could be by stabilizing the assembly of coexpressed Eph receptors and ephrins into lattice-like arrays that span cell-cell contacts and engage both *cis* and *trans* interfaces [23]. However, we did not observe enrichment of EphA3 and ephrin-A3 in regions of cell-cell contact in A549 lung cancer cells coexpressing these proteins (not shown). Furthermore, coexpressed ephrins can block ephrin binding to Eph receptors in *trans* even in the absence of cell-cell contacts [17,20]. Taken together, these data suggest that ephrin binding in *cis* to the fibronectin type III domains of an Eph receptor may promote an additional *cis* interaction between the ephrin-binding pocket of the Eph receptor and the G-H loop of the ephrin. This may occur even when the second interaction is very weak, as in the case of EphA3 and ephrin-B2, since we found that ephrin-B2 coexpression can prevent ephrin-A3 binding to EphA3 in *trans*. A contribution of the Eph receptor ligand-binding domain is also consistent with the trend towards a weaker *cis* association and the weaker attenuation of EphA receptor activation and functional effects observed when interaction between the Eph receptor ligand-binding domain and co-expressed ephrin is prevented (Figure 3 and [18]). However, other possible mechanisms explaining the inhibitory effects of *cis* interactions on Eph receptor activation cannot be excluded, including allosteric conformational changes blocking access to the ephrin-binding pocket of the Eph receptor or intercalation of the ephrin preventing the receptor clustering needed for activation [17,18,20,32].

Previous studies have assumed that Eph receptor-ephrin *cis* interactions exhibit the same A or B class selectivity as *trans* interactions [17,18,20]. However, the fibronectin type III domains of an Eph receptor could conceivably bind a different subset of ephrins than the ligand-binding domain, particularly because the two Eph receptor regions also interact with distinct parts of the ephrins. Our data indeed show that coexpressed ephrin-B2 can strongly inhibit EphA3 interaction with ephrins in *trans* and tyrosine phosphorylation, even though this ephrin does not efficiently bind to the EphA3 ligand-binding domain [25]. However, we could not detect coimmunoprecipitation of ephrin-B2 with EphA3 (data not shown), suggesting that the *cis* association of EphA3 with ephrin-B2 may be weaker than with ephrin-A3 or ephrin-A5 (Figure 3) [18]. Nevertheless, the *cis* inhibition of EphA3 by ephrin-B2 suggests that in at least some cases ephrins that cannot activate a particular Eph receptor can instead inhibit its signaling ability through *cis* association. This represents a novel facet of Eph receptor-ephrin signaling and has functional implications in cancer cells, which can express multiple Eph receptors and ephrins of different classes [44,45] (http://www.broadinstitute.org/ccle/). It will therefore be interesting to investigate the extent of these interclass *cis* interactions and whether this mechanism could explain some puzzling findings. For example, ephrin-B3 knockdown revealed that this ephrin increases EphA2 expression in the U-1810 lung cancer cell line [44]. Since ephrin-B3 is not an activating ligand for EphA2 [25], an explanation for these findings could be that ephrin-B3 interacting in *cis* prevents EphA2 activation and degradation induced by ephrin-A1 in *trans* [9,44,46]. 

Studies in the nervous system have suggested that *cis* interactions are favored under conditions of high ephrin expression, which promotes colocalization of Eph receptors and ephrins in the same plasma membrane microdomains enabling their intermingling [20]. We indeed found that ephrin-A3 overexpressed in lung cancer cells can inhibit EphA2 and EphA3 activation by ephrins in *trans* while overexpression of ephrin-B2 can inhibit activation of EphA3 and EphB4. Importantly, ephrins endogenously expressed at high levels in cancer cells can also participate in inhibitory *cis* interactions, since removal of endogenous GPI-linked ephrin-As from the surface of SKBR3 and MCF7 breast cancer cells with PI-PLC allows increased activation of endogenous EphA2 by soluble ephrin-A1 in *trans*. In contrast, inhibiting the release of GPI-linked ephrin-As through inactivation of matrix metalloproteases in SKBR3 cells did not detectably affect EphA2 activation in *trans* by ephrin-A1 Fc under the conditions of our experiments, presumably due to the already high levels of ephrin-A1 expressed in these cells. It will be interesting to determine whether in cancer cells with moderate ephrin-A levels, inhibiting matrix metalloproteases could enhance the inhibitory effect of *cis* interactions on EphA receptor signaling.

Some of the Eph receptor residues that are predicted to participate in *cis* interaction with ephrins have been reported to be mutated in cancer specimens [23]. We found that the EphA3 G518L lung cancer mutation strengthens the *cis* association of EphA3 with coexpressed ephrin-A3. It will be interesting to examine whether other cancer mutations involving residues predicted to participate in the *cis* interface of other Eph receptors – such as EphA1 R337Q, EphB1 R327H and I332M, and EphB3 E358K in the first fibronectin type III domain as well as EphA5 G547S, EphA6 T493K and R494M, and EphA7 E482D in the second fibronectin type III domain (http://cancer.sanger.ac.uk/cosmic/) – also have functional consequences on *cis* associations with ephrins. 

In summary, our data reveal a signaling mechanism previously uncharacterized in cancer cells whereby ephrin-mediated *cis* attenuation of Eph receptor signaling can inhibit responsiveness to ephrins expressed by other cancer cells or by cells of the tumor microenvironment. Further investigations of the selectivity and functional effects of Eph receptor-ephrin *cis* interactions will provide new information on Eph receptor signaling mechanisms in cancer pathogenesis, which may help the development of new therapeutic approaches.

## Materials and Methods

### Plasmids and lentiviruses

The human EphA3 cDNA was purchased from Invitrogen/Life Technologies (Carlsbad, CA; clone MGC:71556; GenBank accession number NP_005224.2), PCR amplified to include appropriate restriction sites and cloned in pcDNA3. EphA3 was also subcloned into the pLVX-IRES-ZsGreen lentiviral vector (Clontech Laboratories, Mountain View, CA). The truncated versions EphA3 ΔN, comprising a signal peptide followed by amino acids 318-984 of EphA3, was also generated by PCR amplification of full-length EphA3 and cloned in pcDNA3. The EphA3 ΔN G518L mutant was similarly generated by PCR amplification from the previously described full-length EphA3 G518L mutant [14]. Mouse ephrin-A3 cDNA in pcDNA3, including nucleotides 40-744 (GeneBank accession number NM_010108.1), was used as template to generate the ephrin-A3 E129K mutant using the QuickChange Site-Direct mutagenesis kit (Stratagene/Agilent Technologies, La Jolla, CA). The CS-Mm30127-Lv105-ephrin-A3 lentivirus, with mCherry inserted between the signal peptide and the mature coding sequence of mouse ephrin-A3, and the EX-mCHER-Lv105 control lentivirus encoding mCherry were purchased from GeneCopoeia. The mouse mCherry-ephrin-A3 E129K mutant was generated in pcDNA3 using the QuickChange Site-Direct mutagenesis kit and subcloned in the pLVX-IRES-Neo lentiviral vector (Clontech Laboratories). Mouse ephrin-B2 (GeneBank accession number NM_010111.5) with an N-terminal EGFP tag inserted between a signal peptide and the mature coding sequence [47,48] was cloned in the pCCLsin.PPT.hPGK.GFP. pre lentiviral vector [49] replacing the EGFP insert of the vector. The pCCLsin.PPT.hPGK.GFP. pre lentiviral vector encoding EGFP was used as a control. All PCR-amplified and mutated cDNAs were verified by sequencing.

### Cell culture, transfections and infections

The human embryonic kidney (HEK) 293T cell line (ATCC, Manassas, VA), the HEK AD-293 cell line (Cell Biolabs, Inc.), which is a derivative of the HEK 293 cell line with increased adherence, the SKBR3 and MCF7 cell lines (ATCC) were grown in Dulbecco’s Modified Eagle’s Medium (DMEM; Cellgro, Manassas, VA) supplemented with 1 mM L-glutamine, 10% fetal bovine serum, 1 mM sodium pyruvate and antibiotics. The A549 human lung adenocarcinoma and NCI-H226 human squamous cell carcinoma cell lines (ATCC) were grown in Roswell Park Memorial Institute (RPMI) culture medium with the same supplements used for DMEM. 

To activate EphA2 and EphA3 in lung cancer cells, the cells were stimulated for 20 min in complete medium with 2 μg/ml ephrin-A3 Fc fusion protein (R&D Systems, Minneapolis, MN) or Fc (MP Biomedical, Solon, OH) preclustered with 1/10 polyclonal goat anti-human Fc antibody (Jackson ImmunoResearch). To activate EphA2 in breast cancer cells, the cells were stimulated for 20 min with 0.5 µg/ml ephrin-A1 Fc or Fc without preclustering. In addition, some wells were pretreated for 4 hours with 1 U/ml PI-PLC (Invitrogen/Life Technologies) and, in some experiments, some wells were pretreated for 24 hours with 100 μM GM-6001 (stock dissolved in DMSO; Enzo Life Sciences, Farmingdale, NY) or an equivalent DMSO concentration (0.4%) as a control. To activate EphB4, cells were stimulated for 20 min with 2 μg/ml ephrin-B2 Fc preclustered with 6 μg/ml anti-human Fc antibody.

To produce alkaline phosphatase fusion proteins, plasmids encoding EphA3 AP, ephrin-A3 AP, ephrin-A5 AP or ephrin-B2 AP were transiently transfected in HEK293T cells using Lipofectamine 2000 (Invitrogen/Life Technologies) according to the manufacturer’s instructions. Plasmids encoding EphA3, EphA3 ΔN or EphA3 ΔN G518L were transiently transfected in HEK AD-293 cells using Lipofectamine 2000 and the cells were lysed one day after transfection. NCI-H226 and A549 cells were infected with the lentivirus encoding EphA3 and ZsGreen and FACS-sorted. The sorted cells were then infected with lentiviruses encoding mCherry-ephrin-A3 or mCherry and selected with 1 μg/ml puromycin. Alternatively, the sorted cells were infected with lentiviruses encoding EGFP-ephrin-B2 or EGFP. HEK AD-293 cells infected with lentiviruses encoding mCherry-ephrin-A3 or mCherry were selected with puromycin while cells infected with the lentivirus encoding the mCherry-ephrin-A3 E129K mutant were selected with 1.5 mg/ml G418 (Roche Applied Science, Indianapolis, IN).

### Immunoprecipitations, pull-downs and immunoblotting

Cells were washed with cold phosphate-buffered saline (PBS) and lysed in modified RIPA buffer (50 mM Tris-HCl pH 7.6, 150 mM NaCl, 1% Triton X-100, 0.5% sodium deoxycholate, 0.1% SDS, and 2 mM EDTA) or Triton-X100 buffer (50 mM Tris pH 7.5, 150 mM NaCl, 1% Triton, 10% glycerol) with protease and phosphatase inhibitors. The cells were then briefly sonicated.

For immunoprecipitations, cells lysed in modified RIPA buffer were precleared for 15 min at 4 °C with GammaBind Plus sepharose beads and then incubated for 90 min at 4 °C with 2.5 µg anti-EphA2 monoclonal antibody (clone D7; Upstate Biotechnology/Millipore, Lake Placid, NY), anti-EphA3 monoclonal antibody (Invitrogen/Life Technologies), an affinity-purified rabbit polyclonal anti-EphB4 antibody to the human EphB4 C terminal region [50], or anti-dsRed polyclonal antibody (Clontech Laboratories) immobilized on GammaBind Plus sepharose beads (GE Healthcare Bio-Sciences, Piscataway, NJ). For coimmunoprecipitations, cells lysed in Triton X-100 buffer were precleared with GammaBind Plus sepharose beads and then incubated for 3 hours at 4°C with 2.5 µg anti-EphA3 monoclonal antibody immobilized on GammaBind Plus sepharose beads. 

For pull-down of ephrin-A3 from cell culture medium and cell lysates, A549 and H226 cells were grown to confluency in 60 mm plates with 1.5 ml medium for 24 hours (A549 cells) or 48 hours (H226 cells). The conditioned medium was collected and the cells were washed with cold PBS and lysed in 1.5 ml modified RIPA buffer. Culture medium and cell lysates were precleared with GammaBind Plus sepharose beads and then incubated for 1 hour at 4 °C with 1 µg EphA3 Fc (R&D Systems) immobilized on GammaBind Plus sepharose beads. 

Immunoprecipitates, pull-downs and cell lysates were analyzed by immunoblotting with the following antibodies: anti-phosphotyrosine conjugated to horseradish peroxidase (HRP; BD Bioscience, San Jose, CA), anti-EphA3 rabbit polyclonal (sc-919, Santa Cruz Biotechnology, Dallas, TX), anti-EphA2 rabbit polyclonal (Invitrogen/Life Technologies), anti-EphB4 mouse monoclonal (Invitrogen/Life Technologies), anti-ephrin-A1 rabbit monoclonal (Abcam, Cambridge, MA), anti-ephrin-A3 rabbit polyclonal (Santa Cruz Biotechnology), anti-ephrin-A3 chicken immune serum obtained by injecting a mouse ephrin-A3 Fc fusion protein including amino acids 31-213 [45], rabbit anti-human Fc (Jackson ImmunoResearch Laboratories, West Grove, PA), anti-dsRed rabbit polyclonal (Clontech Laboratories, Inc), and anti-GFP rabbit polyclonal (Gentex). Incubation with primary antibodies was followed by incubation with anti-rabbit, anti-mouse or anti-chicken secondary antibodies conjugated to HRP (anti-rabbit and anti-mouse from Millipore, Billerica, MA, and anti-chicken from Sigma-Aldrich, St. Louis, MO) or fluorescently labeled anti-rabbit and anti-mouse secondary antibodies (Odyssey LI-COR, Lincoln, NE). Immunoblots were developed with ECL chemiluminescence HRP detection reagent (GE Healthcare) and the bands quantified using Photoshop. The Odyssey LI-COR system was used for detection in the immunoblots shown in Figure 6, where the bands were quantified with Image Studio Software version 3.1.4.

### Production of AP fusion proteins and AP cell binding assays

Culture medium containing the secreted AP fusion proteins was concentrated using Amicon Ultra Centrifugal filters (Millipore, Billerica, MA) and the concentration of the AP fusion proteins was estimated from AP activity measurements [14]. Assays to measure binding of EphA3 AP or ephrin AP proteins to cells were carried out as previously described [14]. The cells were washed once with cold Hanks’ balanced salt solution (HBAH) containing 0.5 mg/ml bovine serum albumin, 0.1% NaN_3_ and 20 mM HEPES pH 7.0 and then incubated for 90 min with 12 nM of AP fusion protein followed by 6 washes with cold HBAH. The cells were then lysed in 1% Triton X-100, 10 mM Tris-HCl pH 8.0 at room temperature, centrifuged at maximum speed in an Eppendorf benchtop microcentrifuge, and the supernatants were heated at 65°C for 10 min to inactivate endogenous alkaline phosphatase. AP fusion proteins bound to the cells were quantified by measuring the absorbance of the cleaved p-nitrophenyl phosphate chromogenic substrate (Pierce/Thermo Scientific, Rockford, IL). 

### Cell surface biotinylation

To biotinylate cell surface proteins, A549 cells were first kept at 4 °C for 10 min to block endocytosis. The cells were then incubated with 0.5 mg/ml of EZ-link SulfoNHS-LC-Biotin (Pierce/Thermo Scientific) in PBS for 30 min at 4°C, followed by two washes with cold PBS and incubation in quenching buffer (100 mM glycine in PBS) for 14 min at 4 °C. The cells were then lysed in modified RIPA buffer. For quantification of cell surface (biotinylated) EphA3, protein A-coated 96-well plates were incubated with 100 μl anti-EphA3 polyclonal antibody recognizing an epitope in the cytoplasmic region of the receptor (Santa Cruz Biotechnology) at a final concentration of 0.5 µg/ml, washed to remove unbound antibody, then incubated for one hour with cell lysates and washed. EphA3 biotinylation was measured using a streptavidin-HRP conjugate (Pierce/Thermo Scientific) with 2,2'-azino-bis[3-ethylbenzothiazoline-6-sulphonic acid] (ABTS) chromogenic substrate followed by quantification of optical absorbance at 405 nm. For quantification of cell surface ephrin-A3, proteins on the surface of A549 cells were similarly biotinylated. The cells were then lysed in RIPA buffer, mCherry-ephrin-A3 was immunoprecipitated with dsRed antibody, and the immunoprecipitates were probed with a streptavidin-HRP conjugate. 

### Statistical analyses

All statistical analyses were performed with the Program Prism from GraphPad Software (La Jolla, CA).
